# Sharp inequalities for tangent function with applications

**DOI:** 10.1186/s13660-017-1372-5

**Published:** 2017-05-01

**Authors:** Hui-Lin Lv, Zhen-Hang Yang, Tian-Qi Luo, Shen-Zhou Zheng

**Affiliations:** 1 Department of Mathematics, Beijing Jiaotong University, Beijing, 100044 China; 2 Department of Science and Technology, State Grid Zhejiang Electric Power Company Research Institute, Hangzhou, Zhejiang 310014 China

**Keywords:** 33B10, 26D05, trigonometric function, inequalities, Sine integral, Catalan constant

## Abstract

In the article, we present new bounds for the function $e^{t\cot(t)-1}$ on the interval $(0, \pi/2)$ and find sharp estimations for the Sine integral and the Catalan constant based on a new monotonicity criterion for the quotient of power series, which refine the Redheffer and Becker-Stark type inequalities for tangent function.

## Introduction

The study of this paper is concerned with the following inequality:

1.1 $$ \frac{\sin t}{t}\geq\frac{\pi^{2}-t^{2}}{\pi^{2}+t^{2}}, \quad t\in ( 0,\pi ), $$

which was posted by Redheffer in [1] and was proved by Williams [2]. Recently, Zhu and Sun [3] extended the Redheffer inequality (1.1) to the tangent function, and they established the following inequalities:

1.2 $$ \biggl( \frac{\pi^{2}+4t^{2}}{\pi^{2}-4t^{2}} \biggr) ^{\pi^{2}/24}< \frac {\tan t}{t}< \frac{\pi^{2}+4t^{2}}{\pi^{2}-4t^{2}}, \quad t\in ( 0,\pi/2 ) $$

with the best exponents $\pi^{2}/24$ and 1. Zhu [4] further refined the double inequality

1.3 $$ \biggl( \frac{\sqrt{\pi^{4}+48t^{4}}}{\pi^{2}-4t^{2}} \biggr) ^{1/2}< \frac{\tan t}{t}< \biggl( \frac{\sqrt{\pi^{4}+48t^{4}}}{\pi^{2}-4t^{2}} \biggr) ^{\pi^{2}/6},\quad t\in ( 0,\pi/2 ). $$

It is worth noting that Becker and Stark [5] in 1978 showed the double inequality

1.4 $$ \frac{8}{\pi^{2}-4t^{2}}< \frac{\tan t}{t}< \frac{\pi^{2}}{\pi ^{2}-4t^{2}}, \quad t\in ( 0,\pi/2 ), $$

where 8 and $\pi^{2}$ are the best constants. Later, Zhu and Hua [6] gave a general refinement of the Becker-Stark inequalities (1.4) by the power series expansion of the tangent function in terms of the Bernoulli numbers. In particular, they proved that for $t\in ( 0,\pi/2 ) $ the double inequality

1.5 $$ \frac{\pi^{2}+4 ( 8/\pi^{2}-1 ) t^{2}}{\pi^{2}-4t^{2}}< \frac {\tan t}{t}< \frac{\pi^{2}+ ( \pi^{2}/3-4 ) t^{2}}{\pi^{2}-4t^{2}} $$

holds with the best constants $4 ( 8/\pi^{2}-1 ) $ and $( \pi^{2}/3-4 ) $; also see [7]. Chen and Cheung [8] further presented an improvement of the left hand side inequality in (1.4), which states that

1.6 $$ \biggl( \frac{\pi^{2}}{\pi^{2}-4t^{2}} \biggr) ^{\alpha}< \frac{\tan t}{t}< \biggl( \frac{\pi^{2}}{\pi^{2}-4t^{2}} \biggr) ^{\beta} $$

holds for $t\in ( 0,\pi/2 ) $ with the best exponents $\alpha =\pi^{2}/12$ and $\beta=1$ (also cf. [9]). Another improvement involving the left hand side one in (1.4) was made in [10] by Nishizawa. Very recently, Bhayo and Sándor [11], Corollary 3, again proved the Becker-Stark inequalities (1.4) by using Redheffer inequality (1.1), which reveals the implicit relation between Redheffer’s and Becker-Stark’s inequalities. They in [11], Corollaries 2, also stated that for $t\in ( 0,\pi/2 ) $ we have

1.7 $$ \frac{\pi^{2}-4t^{2}-\pi^{2}t^{2}/3}{\pi^{2}-t^{2}}< \frac{t}{\tan t}< \frac {\pi^{2}-4t^{2}}{\pi^{2}-t^{2}}. $$

It is an important observation that Yang *et al.* [12], (93), in 2014 considered the bounds for function $e^{t\cot t-1}$ and established a number of inequalities for trigonometric functions. In particular, they in [12], Corollary 16, showed that for $t\in ( 0,\pi/2 ) $

$$e^{-4t^{2}/\pi^{2}}< e^{t\cot t-1}< e^{-t^{2}/3},$$

which can be written as

1.8 $$ 1-\frac{4t^{2}}{\pi^{2}}< \frac{t}{\tan t}< 1-\frac{t^{2}}{3}. $$

Inspired by these results mentioned above, the aim of this paper is to determine the best bounds for $Y ( t ) =e^{t\cot t-1}$ in terms of

1.9 $$ B_{p} ( t ) =\left \{ \textstyle\begin{array}{l@{\quad}l} ( 1-pt^{2} ) ^{1/ ( 3p ) } & \text{if }p\in ( -\infty,0 ) \cup( 0,4/\pi^{2} ] ,\\ e^{-t^{2}/3} & \text{if }p=0, \end{array}\displaystyle \right . $$

on $( 0,\pi/2 ) $, that is to say, we will determine the best parameters $p,q\in(-\infty, 4/\pi^{2} ]$ such that the double inequality

1.10 $$ \bigl( 1-pt^{2} \bigr) ^{1/ ( 3p ) }< \exp \biggl( \frac {t}{\tan t}-1 \biggr) < \bigl( 1-qt^{2} \bigr) ^{1/ ( 3q ) } $$

holds for all $t\in ( 0,\pi/2 ) $. Inequalities (1.10) also can be rewritten as

$$1+\frac{1}{3p}\ln \bigl( 1-pt^{2} \bigr) < \frac{t}{\tan t}< 1+ \frac{1}{3q} \ln \bigl( 1-qt^{2} \bigr) , $$

which offers a new type of bounds being different of the previous papers for the tangent function.

## Some useful lemmas

In order to prove the main Theorem 1 in the next section, we need some preliminary lemmas. To this end, we first introduce a useful auxiliary function $H_{f,g}$. For $-\infty\leq a< b\leq\infty$, let *f* and *g* be differentiable on $(a,b)$ and $g^{\prime}\neq0$ on $(a,b)$. Then the function $H_{f,g}$ is defined by

2.1 $$ H_{f,g}:=\frac{f^{\prime}}{g^{\prime}}g-f. $$

The function $H_{f,g}$ has been investigated with some well properties in [13], Properties 1, 2, which plays an important role in the proof of a monotonicity criterion for the quotient of power series; also see [14].

### Lemma 1

[14], Theorem 2.1, [15], Lemma 3.1, and [16], Lemma 1.1

*Let* $A ( t ) =\sum_{k=0}^{\infty}a_{k}t^{k}$ *and* $B ( t ) =\sum_{k=0}^{\infty }b_{k}t^{k}$ *be two real power series converging on* $( -r,r ) $ *and* $b_{k}>0$ *for all* *k*. *Suppose that*, *for certain* $m\in \mathbb{N} $, *the non*-*constant sequence* $\{ a_{k}/b_{k} \} $ *is increasing* (*resp*. *decreasing*) *for* $0\leq k\leq m$ *and decreasing* (*resp*. *increasing*) *for* $k\geq m$. *Then the function* $A/B$ *is strictly increasing* (*resp*. *decreasing*) *on* $( 0,r ) $ *if and only if* $H_{A,B} ( r^{-} ) \geq \textit{(resp. $\leq$) }0$. *Moreover*, *if* $H_{A,B} ( r^{-} ) < \textit{(resp. $>$) }0$, *then there exists* $t_{0}\in ( 0,r ) $ *such that the function* $A/B$ *is strictly increasing* (*resp*. *decreasing*) *on* $( 0,t_{0} ) $ *and strictly decreasing* (*resp*. *increasing*) *on* $( t_{0},r ) $.

### Lemma 2

[17], p. 75

*Let* $0< t<\pi$. *Then*

2.2 $$\begin{aligned}& \cot t =\frac{1}{t}-\sum_{n=1}^{\infty} \frac{2^{2n}}{ ( 2n ) !}|B_{2n}|t^{2n-1}, \end{aligned}$$

2.3 $$\begin{aligned}& \frac{1}{\sin^{2}t} =\frac{1}{t^{2}}+\sum_{n=1}^{\infty} \frac{ ( 2n-1 ) 2^{2n}}{ ( 2n ) !}|B_{2n}|t^{2n-2}, \end{aligned}$$

*where* $B_{n}$ *is the Bernoulli number*.

### Lemma 3

[18]

*Let* $B_{2n}$ *and* $B_{2n-2}$ *be the even*-*indexed Bernoulli numbers*. *Then*

2.4 $$ \frac{1}{ ( 2\pi ) ^{2}}\frac{2n ( 2n-1 ) ( 2^{2n-3}-1 ) }{2^{2n-3}}< \frac{ \vert B_{2n} \vert }{ \vert B_{2n-2} \vert }< \frac{1}{ ( 2\pi ) ^{2}} \frac{2n ( 2n-1 ) 2^{2n-1}}{2^{2n-1}-1}. $$

*Consequently*, *we have*

$$\frac{n ( 2n-1 ) }{ ( n+1 ) ( 2n+1 ) }\frac{ ( 2^{2n+1}-1 ) ( 2^{2n-3}-1 ) }{2^{4n-2}}< \frac{ \vert B_{2n} \vert ^{2}}{ \vert B_{2n-2}B_{2n+2} \vert }< \frac{n ( 2n-1 ) }{ ( 2n+1 ) ( n+1 ) }\frac{2^{4n-2}}{ ( 2^{2n-1}-1 ) ^{2}}.$$

### Lemma 4

*Let the function* *g* *be defined on* $( 0,\pi ) $ *by*

2.5 $$ g ( t ) =\frac{3t-3\cos t\sin t-2t\sin^{2}t}{3t^{2} ( t-\sin t\cos t ) }. $$

*Then* *g* *is strictly increasing from* $( 0,\pi/2 ) $ *onto* $( 2/15,4/ ( 3\pi^{2} ) ) $ *and decreasing from* $( \pi/2,\pi ) $ *onto* $( 1/\pi^{2},4/ ( 3\pi^{2} ) ) $.

### Proof

To avoid complicated calculations, we here make use of Lemmas 1, 2 and 3 to prove this lemma. For this purpose, we write $g ( t ) $ as

$$g ( t ) =\frac{\frac{t}{\sin^{2}t}-\frac{\cos t}{\sin t}-\frac {2}{3}t}{\frac{t^{3}}{\sin^{2}t}-t^{2}\frac{\cos t}{\sin t}}:=\frac {g_{1} ( t ) }{g_{2} ( t ) }, $$

then applying Lemma 2 yields

$$g ( t ) =\frac{g_{1} ( t ) }{g_{2} ( t ) }=\frac{\sum_{n=1}^{\infty}\frac{2^{2n+2}}{ ( 2n+1 ) !} \vert B_{2n+2} \vert t^{2n-2}}{\sum_{n=1}^{\infty}\frac{2^{2n}}{ ( 2n-1 ) !} \vert B_{2n} \vert t^{2n-2}}:=\frac{\sum_{n=0}^{\infty}a_{n+1}x^{n}}{\sum_{n=1}^{\infty}b_{n+1}x^{n}} ,$$

where

$$a_{n}=\frac{2^{2n+2}}{ ( 2n+1 ) !} \vert B_{2n+2} \vert ,\qquad b_{n}=\frac{2^{2n}}{ ( 2n-1 ) !} \vert B_{2n} \vert ,\qquad x=t^{2}\in \bigl( 0,\pi^{2} \bigr) . $$

We now prove that the sequence $\{a_{n}/b_{n}\}$ is increasing for $1\leq n\leq2$ and decreasing for $n\geq2$. A simple check yields

$$\frac{a_{1}}{b_{1}}=\frac{2}{15}>\frac{a_{2}}{b_{2}}=\frac{1}{7}< \frac {a_{3}}{b_{3}}=\frac{2}{15}< \frac{a_{4}}{b_{4}}=\frac{25}{198}, $$

and it remains to show that $a_{n-1}/b_{n-1}>a_{n}/b_{n}$ for $n\geq4$. Indeed, we have

$$\begin{aligned} \frac{a_{n-1}}{b_{n-1}} \Big/ \frac{a_{n}}{b_{n}} & = \frac{2}{ ( n-1 ) ( 2n-1 ) } \frac{ \vert B_{2n} \vert }{ \vert B_{2n-2} \vert } \Big/ \biggl( \frac {2}{n ( 2n+1 ) }\frac{ \vert B_{2n+2} \vert }{ \vert B_{2n} \vert } \biggr) \\ & =\frac{n ( 2n+1 ) }{ ( 2n-1 ) ( n-1 ) }\frac{ \vert B_{2n} \vert }{ \vert B_{2n-2} \vert }\frac{ \vert B_{2n} \vert }{ \vert B_{2n+2} \vert }. \end{aligned}$$

Then by Lemma 3, we get

$$\begin{aligned} \frac{a_{n-1}}{b_{n-1}} \Big/ \frac{a_{n}}{b_{n}}-1 & >\frac{n ( 2n+1 ) }{ ( 2n-1 ) ( n-1 ) }\frac{n ( 2n-1 ) }{ ( n+1 ) ( 2n+1 ) }\frac{ ( 2^{2n+1}-1 ) ( 2^{2n-3}-1 ) }{2^{4n-2}}-1 \\ & =\frac{n^{2}}{n^{2}-1}\frac{ ( 2^{2n+1}-1 ) ( 2^{2n-3}-1 ) }{2^{4n-2}}-1 \\ & =\frac{ ( 2^{2n+1}-17n^{2} ) 2^{2n}+8n^{2}}{2\times 2^{4n} ( n-1 ) ( n+1 ) }>0\quad\text{for }n\geq4, \end{aligned}$$

where the inequality holds due to $2^{2n+1}-17n^{2}>0$ for $n\geq4$. This proves the piecewise monotonicity of $\{ a_{n}/b_{n} \} _{n\geq1}$.

According to Lemma 1, we also have to check that $H_{g_{1},g_{2}} ( \pi^{-} ) <0$ and $H_{g_{1},g_{2}} ( \pi/2 ) =0$. In fact, we have

$$\begin{aligned} H_{g_{1},g_{2}} ( \sqrt{x} ) & =\frac{g_{1}^{\prime} ( \sqrt{x} ) }{g_{2}^{\prime} ( \sqrt{x} ) }g_{2} ( \sqrt{x} ) -g_{1} ( \sqrt{x} ) \\ & =\frac{ ( \frac{t}{\sin^{2}t}-\frac{\cos t}{\sin t}-\frac{2}{3}t ) ^{\prime}}{ ( \frac{t^{3}}{\sin^{2}t}-t^{2}\frac{\cos t}{\sin t} ) ^{\prime}} \biggl( \frac{t^{3}}{\sin^{2}t}-t^{2} \frac {\cos t}{\sin t} \biggr) - \biggl( \frac{t}{\sin^{2}t}-\frac{\cos t}{\sin t}- \frac {2}{3}t \biggr) \\ & =\frac{1}{3\sin t}\frac{2t^{3}\cos t\sin t+3t^{2}\cos^{2}t+t ( \sin^{2}t-6 ) \sin t\cos t+3\cos^{2}t\sin^{2}t}{t^{2}\cos t-2t\sin t+\cos t\sin^{2}t} \\ & \rightarrow-\infty\quad \text{as }t\rightarrow\pi^{-}, \end{aligned}$$

then Lemma 1 leads to the result that there is a unique $t_{0}\in ( 0,\pi ) $ such that *g* is increasing on $( 0,t_{0} ) $ and decreasing on $( t_{0},\pi ) $. Note that

$$g^{\prime} ( t ) =\frac{g_{2}^{\prime} ( t ) }{g_{2} ( t ) ^{2}}H_{g_{1},g_{2}} ( t ) = \frac {g_{2}^{\prime} ( t ) }{g_{2} ( t ) ^{2}} \biggl[ \frac{g_{1}^{\prime} ( t ) }{g_{2}^{\prime} ( t ) }g_{2} ( t ) -g_{1} ( t ) \biggr] \rightarrow0\quad \text{as }t\rightarrow\pi/2, $$

we clearly see that the unique $t_{0}=\pi/2$. A simple computation yields

$$g \bigl( 0^{+} \bigr) =\frac{2}{15}, \qquad g \biggl( \frac{\pi}{2} \biggr) =\frac{4}{3\pi^{2}}, \qquad g \bigl( \pi^{-} \bigr) =\frac{1}{\pi^{2}}, $$

which completes the proof. □

### Remark 1

If we use an ordinary method to prove the piecewise monotonicity of *g*, then it is very troublesome. For example, a direct computation yields

$$g ( t ) =\frac{1}{3}\frac{4t-3\sin2t+2t\cos2t}{t^{2} ( 2t-\sin2t ) }\overset{u=2t\in ( 0,2\pi ) }{=}\frac{4}{3}\frac{2u-3\sin u+u\cos u}{u^{2} ( u-\sin u ) }:=g_{1} ( u ) , $$

then differentiating $g_{1} ( u ) $ gives

$$g_{1}^{\prime} ( u ) =-\frac{4}{3}\frac{u^{3}\sin u+3 ( 1+\cos u ) u^{2}-u ( 11+\cos u ) \sin u+6\sin^{2}u}{u^{3} ( u-\sin u ) ^{2}} .$$

As a result, there are various approaches to showing the piecewise monotonicity of $g_{1}$ on $( 0,2\pi ) $, but it seems to be difficult. It thus can be seen that our method used previously is relatively easy.

### Lemma 5

*For* $t\in ( 0,\pi/2 ) $, *let* $p\longmapsto B_{p} ( t ) $ *and* $p\longmapsto\alpha_{p} ( t ) $ *be respectively defined on* $(-\infty,4/\pi^{2}]$ *by* (1.9) *and*

2.6 $$ \alpha_{p} ( t ) =\frac{\exp ( t\cot t-1 ) }{ ( 1-pt^{2} ) ^{1/ ( 3p ) }} \quad\textit{if }p\neq0,\quad\textit{and}\quad \alpha_{0} ( t ) =\exp \biggl( t\cot t-1+ \frac{t^{2}}{3} \biggr) . $$

*Then* $p\longmapsto B_{p} ( t ) $ *and* $p\longmapsto\alpha _{p} ( \pi/2 ) B_{p} ( t ) $ *are strictly decreasing and increasing on* $(-\infty,4/\pi^{2}]$, *respectively*. *Moreover*, *there is a unique* $p_{0}\approx0.13484$ *such that* $\alpha_{p} ( \pi/2 ) <1$ *for* $p\in ( -\infty,p_{0} ) $ *and* $\alpha_{p} ( \pi/2 ) >1$ *for* $p\in ( p_{0},4/\pi^{2} ) $, *where* $p_{0}$ *is the unique solution of the equation* $\alpha_{p} ( \pi/2 ) =1$ *on* $( -\infty,4/\pi^{2} ) $.

### Proof

Let $p\neq0$. Logarithmic differentiation yields

$$\begin{aligned}& \frac{\partial\ln B_{p} ( t ) }{\partial p}=-\frac{1}{3p^{2}}r ( t ) ,\\& \frac{\partial\ln [ \alpha_{p} ( \pi/2 ) B_{p} ( t ) ] }{\partial p}=\frac{1}{3p^{2}} \biggl[ r \biggl( \frac{\pi }{2} \biggr) -r ( t ) \biggr] , \end{aligned}$$

where

$$r ( t ) =\ln \bigl( 1-pt^{2} \bigr) +\frac{1}{1-pt^{2}}-1. $$

Differentiation again leads to

$$r^{\prime} ( t ) =\frac{2p^{2}t^{3}}{ ( 1-pt^{2} ) ^{2}}>0 $$

for $t\in ( 0,\pi/2 ) $, which means that $0=r ( 0^{+} ) < r ( t ) < r ( \pi/2 ) $. These together with $B_{0} ( t ) =\lim_{p\rightarrow0}B_{p} ( t ) $ and $\alpha_{0} ( t ) =\lim_{p\rightarrow0}\alpha_{p} ( t ) $ show that $p\longmapsto B_{p} ( t ) $ and $p\longmapsto\alpha _{p} ( ( \pi/2 ) ) B_{p} ( t ) $ are strictly decreasing and increasing on $(-\infty,4/\pi^{2}]$, respectively.

Note the increasing property of $p\longmapsto\ln\alpha_{p} ( \pi /2 ) =-1-\ln B_{p} ( \pi/2 ) $ on $(-\infty,4/\pi^{2}]$ and

$$\begin{aligned}& \ln\alpha_{0} ( \pi/2 ) =\lim_{p\rightarrow0} \biggl[ -1- \frac{1}{3p}\ln \biggl( 1-\frac{p\pi^{2}}{4} \biggr) \biggr] = \frac{\pi ^{2}}{12}-1< 0, \\& \ln\alpha_{1/3} ( \pi/2 ) =-1-\ln \biggl( 1-\frac{\pi^{2}}{12} \biggr) >0, \end{aligned}$$

which implies that there is a unique $p_{0}\in ( 0,1/3 ) $ such that $\ln\alpha_{p}<0$ for $p\in ( -\infty,p_{0} ) $ and $\alpha_{p}>1$ for $p\in ( p_{0},4/\pi^{2} ) $. Solving the equation $\ln \alpha_{p} ( \pi/2 ) =0$ for *p* gives $p=p_{0}\approx0.13484$. The proof is finished. □

## Main results

This section is devoted to stating and proving the main results concerning some inequalities for the tangent function. More precisely, we have the following.

### Theorem 1

*For* $p\in(-\infty,4/\pi^{2}]$, *let* $Y ( t ) =\exp ( t\cot t-1 ) $ *and* $B_{p} ( t ) $ *be defined on* $( 0,\pi/2 ) $ *by* (1.9).

(i) *If* $p\leq2/15\approx0.13333$, *then the function* $t\longmapsto Y ( t ) /B_{p} ( t ) $ *is strictly decreasing on the interval* $( 0,\pi/2 ) $. *Consequently*, *for all* $t\in ( 0,\pi/2 ) $
  3.1 $$ \alpha_{p} ( \pi/2 ) \bigl( 1-pt^{2} \bigr) ^{1/ ( 3p ) }< \exp \biggl( \frac{t}{\tan t}-1 \biggr) < \bigl( 1-pt^{2} \bigr) ^{1/ ( 3p ) } $$
  *with the best coefficients* 1 *and* $\alpha_{p} ( \pi/2 ) $ *defined by* (2.6).
(ii) *If* $p\geq4/ ( 3\pi^{2} ) \approx0.13509$, *then the function* $t\longmapsto Y ( t ) /B_{p} ( t ) $ *is strictly increasing on* $( 0,\pi/2 ) $, *and therefore*, *for all* $t\in ( 0,\pi/2 ) $,
  3.2 $$ \bigl( 1-pt^{2} \bigr) ^{1/ ( 3p ) }< \exp \biggl( \frac{t}{\tan t}-1 \biggr) < \alpha_{p} ( \pi/2 ) \bigl( 1-pt^{2} \bigr) ^{1/ ( 3p ) } $$
  *with the best coefficients* 1 *and* $\alpha_{p} ( \pi/2 ) $ *defined by* (2.6).
(iii) *If* $2/15< p<4/ ( 3\pi^{2} ) $, *then there is a* $t_{0} \in ( 0,\pi/2 ) $ *such that the function* $t\longmapsto Y ( t ) /B_{p} ( t ) $ *is strictly increasing on* $( 0,t_{0} ) $ *and decreasing on* $( t_{0},\pi/2 ) $, *and hence*, *for all* $t\in ( 0,\pi/2 ) $,
  3.3 $$ \min \bigl( \alpha_{p} ( \pi/2 ) ,1 \bigr) \bigl( 1-pt^{2} \bigr) ^{1/ ( 3p ) }< \exp \biggl( \frac{t}{\tan t}-1 \biggr) < \beta_{p} \bigl( 1-pt^{2} \bigr) ^{1/ ( 3p ) } $$
  *with*
  $$\beta_{p}=\frac{t_{0}\cot t_{0}-1}{ ( 1-pt_{0}^{2} ) ^{1/ ( 3p ) }}, $$
  *where* $t_{0}$ *is the unique solution of the equation*
  3.4 $$ \frac{\cos t}{\sin t}-\frac{t}{\sin^{2}t}+\frac{2t}{3 ( 1-pt^{2} ) }=0 $$
  *on* $( 0,\pi/2 ) $.

*When* $p\in{}[ p_{0},4/ ( 3\pi^{2} )) $, *the double inequality* (3.2) *still holds for all* $t\in ( 0,\pi/2 ) $. *In particular*, *when* $p=p_{0}$, *we have*

3.5 $$ \bigl( 1-p_{0}t^{2} \bigr) ^{1/ ( 3p_{0} ) }< \exp \biggl( \frac {t}{\tan t}-1 \biggr) < \beta_{p_{0}} \bigl( 1-p_{0}t^{2} \bigr) ^{1/ ( 3p_{0} ) } $$

*with the best constants* 1 *and* $\beta_{p_{0}}\approx1.0002$.

### Proof

Let

3.6 $$ f ( t ) =\ln Y ( t ) -\ln B_{p} ( t ) = \left\{ \textstyle\begin{array}{l@{\quad}l}\frac{t}{\tan t}-1-\frac{1}{3p}\ln ( 1-pt^{2} ) & \text{if }p\neq0,\\ \frac{t}{\tan t}-1+\frac{t^{2}}{3} & \text{if }p=0. \end{array}\displaystyle \right . $$

Differentiation yields

$$\begin{aligned} f^{\prime} ( t ) & =\frac{\cos t}{\sin t}-\frac{t}{\sin^{2}t}+ \frac{2t}{3 ( 1-pt^{2} ) } \\ & =\frac{3t^{2} ( t-\cos t\sin t ) }{3 ( 1-pt^{2} ) \sin^{2}t} \biggl( p-\frac{3t-3\cos t\sin t-2t\sin^{2}t}{3t^{2} ( t-\sin t\cos t ) } \biggr) \\ & =\frac{3t^{2} ( t-\cos t\sin t ) }{3 ( 1-pt^{2} ) \sin^{2}t} \bigl[ p-g ( t ) \bigr] , \end{aligned}$$

where $g ( t ) $ is defined by (2.5).

Noticing that $( t-\cos t\sin t ) = [ 2t-\sin ( 2t ) ] /2>0$ for $t\in ( 0,\pi/2 ) $ and $( 1-pt^{2} ) >0$ for $p\in(-\infty,4/\pi^{2}]$ and $t\in ( 0,\pi/2 ) $, we easily see that, for all $t\in ( 0,\pi/2 ) $,

3.7 $$ \operatorname{sgn}f^{\prime} ( t ) =\operatorname{sgn} \bigl( p-g ( t ) \bigr) . $$

As shown in Lemma 4, the function *g* is strictly increasing from $( 0,\pi/2 ) $ onto $( 2/15,4/ ( 3\pi^{2} ) ) $. We are now in a position to distinguish three cases to prove the required result.

*Case 1*: $p\leq\min_{t\in ( 0,\pi/2 ) }g ( t ) =2/15$. Then we obtain $f^{\prime} ( t ) \leq0$ for $t\in ( 0,\pi/2 ) $, which means that *f* is strictly decreasing on $( 0,\pi/2 ) $. Consequently, we can deduce the following observation:

$$\ln\alpha_{p} \biggl( \frac{\pi}{2} \biggr) =f \biggl( \frac{\pi }{2}^{-} \biggr) < f ( t ) < f \bigl( 0^{+} \bigr) =0, $$

which is equivalent to the double inequality (3.1) holding for all $t\in ( 0,\pi/2 ) $ with the best coefficients 1 and $\alpha_{p}$.

*Case 2*: $p\geq\max_{t\in ( 0,\pi/2 ) }g ( t ) =4/ ( 3\pi^{2} ) $. Similarly, we have $f^{\prime} ( t ) >0$ for $t\in ( 0,\pi/2 ) $, which implies that the double inequality (3.2) holds for all $t\in ( 0,\pi/2 ) $ with the best coefficients 1 and $\alpha_{p}( \pi/2 ) $.

*Case 3*: $2/15< p<4/ ( 3\pi^{2} ) $. Since $t\longmapsto p-g ( t ) :=g_{1} ( t ) $ is strictly decreasing on $( 0,\pi/2 ) $ with

$$g_{1} \bigl( 0^{+} \bigr) =p-\frac{2}{15}>0\quad\text{and}\quad g_{1} \biggl( \frac {\pi}{2}^{-} \biggr) =p- \frac{4}{3\pi^{2}}< 0, $$

we find that there is $t_{0}\in ( 0,\pi/2 ) $ such that $g_{1} ( t ) >0$ for $t\in ( 0,t_{0} ) $ and $g_{1} ( t ) <0$ for $t\in ( t_{0},\pi/2 ) $. This indicates that *f* is strictly increasing on $( 0,t_{0} ) $ and decreasing on $( t_{0},\pi/2 ) $. Therefore, we deduce that

$$\min \bigl( 0,\ln\alpha_{p} ( \pi/2 ) \bigr) =\min \biggl( f \bigl( 0^{+} \bigr) , f \biggl( \frac{\pi}{2}^{-} \biggr) \biggr) < f ( t ) \leq f ( t_{0} ) =\ln\beta_{p}$$

for all $t\in ( 0,\pi/2 ) $, that is, (3.3) holds for all $t\in ( 0,\pi/2 ) $, where $\beta_{p}=Y ( t_{0} ) /B_{p} ( t_{0} ) $.

When $p\in{}[ p_{0},4/ ( 3\pi^{2} )) $, by Lemma 5 we have $\alpha_{p}\geq1$, and it follows that

$$0< f ( t ) \leq f ( t_{0} ) =\ln\beta_{p}, $$

that is, the double inequality (3.2) still holds for $t\in ( 0,\pi/2 ) $.

In particular, for $p=p_{0}\approx0.13484$, solving equation (3.4) for *t* yields $t_{0}\approx1.26254$, and hence $\beta_{p_{0}}\approx1.0002$. Thus we complete the proof. □

Taking $p=2/15\approx0.13333, 1/8, 1/9, 0,\text{ and }\rightarrow -\infty$, respectively. Then by part (i) of Theorem 1 and the monotonicity of $p\longmapsto B_{p} ( t ) $ and $p\longmapsto\alpha_{p} ( \pi/2 ) B_{p} ( t ) $ given in Lemma 5, we immediately obtain the following conclusion.

### Corollary 1

*For* $t\in ( 0,\pi/2 ) $, *the inequalities*

3.8 $$\begin{aligned} e^{-1} & < \alpha_{0}e^{-t^{2}/3}< \alpha_{1/9} \biggl( 1-\frac{t^{2}}{9} \biggr) ^{3}< \alpha_{1/8} \biggl( 1-\frac{t^{2}}{8} \biggr) ^{8/3} \\ & < \alpha_{2/15} \biggl( 1-\frac{2t^{2}}{15} \biggr) ^{5/2}< \exp \biggl( \frac{t}{\tan t}-1 \biggr) < \biggl( 1-\frac{2t^{2}}{15} \biggr) ^{5/2} \\ & < \biggl( 1-\frac{t^{2}}{8} \biggr) ^{8/3}< \biggl( 1- \frac{t^{2}}{9} \biggr) ^{3}< e^{-t^{2}/3}< 1 \end{aligned}$$

*holds with the best coefficients*

$$\begin{aligned}& \alpha_{2/15}=e^{-1} \biggl( 1-\frac{\pi^{2}}{30} \biggr) ^{-5/2}\approx0.99742, \qquad \alpha_{1/8}=e^{-1} \biggl( 1-\frac{\pi^{2}}{32} \biggr) ^{-8/3}\approx0.98356,\\& \alpha_{1/9}=e^{-1} \biggl( 1-\frac{\pi^{2}}{36} \biggr) ^{-3}\approx 0.96200, \qquad \alpha_{0}=e^{\pi^{2}/12-1}\approx0.83733. \end{aligned}$$

Likewise, taking $p=4/ ( 3\pi^{2} ) \approx0.13509, 1/7, 1/6, 1/3,\text{ and }4/\pi^{2}$, respectively, we have the following.

### Corollary 2

*For* $t\in ( 0,\pi/2 ) $, *the inequalities*

3.9 $$\begin{aligned} \biggl( 1-\frac{4t^{2}}{\pi^{2}} \biggr) ^{3\pi^{2}/4} & < \biggl( 1- \frac{t^{2}}{3} \biggr) < \biggl( 1-\frac{t^{2}}{6} \biggr) ^{2}< \biggl( 1-\frac{t^{2}}{7} \biggr) ^{7/3} \\ &< \biggl( 1-\frac{4t^{2}}{3\pi^{2}} \biggr) ^{\pi^{2}/4} < \exp \biggl( \frac{t}{\tan t}-1 \biggr) < \alpha_{4/ ( 3\pi^{2} ) } \biggl( 1-\frac{4t^{2}}{3\pi^{2}} \biggr) ^{\pi^{2}/4} \\ &< \alpha_{1/7} \biggl( 1-\frac{t^{2}}{7} \biggr) ^{7/3} < \alpha_{1/6} \biggl( 1-\frac{t^{2}}{6} \biggr) ^{2}< \alpha_{1/3} \biggl( 1-\frac{t^{2}}{3} \biggr) \end{aligned}$$

*hold with the best coefficients*

$$\begin{aligned}& \alpha_{4/ ( 3\pi^{2} ) }=e^{-1} \biggl( \frac{3}{2} \biggr) ^{\pi^{2}/4}\approx1.0004, \qquad \alpha_{1/7}=e^{-1} \biggl( 1-\frac {\pi^{2}}{28} \biggr) ^{-7/3}\approx1.0142,\\& \alpha_{1/6}=e^{-1} \biggl( 1-\frac{\pi^{2}}{24} \biggr) ^{-2}\approx 1.0613, \qquad \alpha_{1/3}=e^{-1} \biggl( 1-\frac{\pi^{2}}{12} \biggr) ^{-1}\approx2.0722. \end{aligned}$$

### Theorem 2

*Let* $p,q\in ( -\infty,4/\pi^{2} ] $. *Then the double inequality*

3.10 $$ \bigl( 1-pt^{2} \bigr) ^{1/ ( 3p ) }< \exp \biggl( \frac {t}{\tan t}-1 \biggr) < \bigl( 1-qt^{2} \bigr) ^{1/ ( 3q ) } $$

*holds for all* $t\in ( 0,\pi/2 ) $ *if and only if* $p\geq p_{0}\approx0.13484$ *and* $q\leq2/15\approx0.13333$, *where* $p_{0}$ *is defined in Lemma* 5.

### Proof

Clearly, the sufficiency easily follows by Theorem 1. The necessary condition for the right hand side inequality in (3.10) to hold for $t\in ( 0,\pi/2 ) $ follows from the limit relation

$$\lim_{t\rightarrow0^{+}}\frac{\ln Y ( t ) -\ln B_{q} ( t ) }{t^{4}}=\frac{1}{90} ( 15q-2 ) \leq0. $$

The necessary condition for the left hand side inequality in (3.10) to hold for $t\in ( 0,\pi/2 ) $ can be obtained from the inequality

$$\lim_{t\rightarrow\pi/2}\frac{Y ( t ) }{B_{p} ( t ) }=e^{-1} \biggl( 1- \frac{p\pi^{2}}{4} \biggr) ^{-1/ ( 3p ) }=\alpha_{p}\geq1. $$

It follows from Lemma 5 that $p\geq p_{0}$, which completes the proof. □

## Comparisons and remarks

By Theorem 2, we have

4.1 $$ 1+\frac{1}{3p_{0}}\ln \bigl( 1-p_{0}t^{2} \bigr) < \frac{t}{\tan t}< 1+\frac {5}{2}\ln \biggl( 1-\frac{2}{15}t^{2} \biggr) , $$

where $p_{0}\approx0.13484$.

We denote the lower bounds for $t/\tan t$ given in the inequalities (4.1), (1.2), (1.3), (1.4), (1.5), and (1.7), respectively, by

$$\begin{aligned}& LY ( t ) =1+\frac{1}{3p_{0}}\ln \bigl( 1-p_{0}t^{2} \bigr) , \qquad ZS ( t ) =\frac{\pi^{2}-4t^{2}}{\pi^{2}+4t^{2}},\qquad Z ( t ) = \biggl( \frac{\pi^{2}-4t^{2}}{\sqrt{\pi^{4}+48t^{4}}} \biggr) ^{\pi^{2}/6}, \\& BS_{1} ( t ) =1-\frac{4}{\pi^{2}}t^{2},\qquad ZH ( t ) =\frac{\pi^{2}-4t^{2}}{\pi^{2}+ ( \pi^{2}/3-4 ) t^{2}}, \qquad BS_{2} ( t ) =\frac{\pi^{2}-4t^{2}-\pi ^{2}t^{2}/3}{\pi^{2}-t^{2}}. \end{aligned}$$

### Proposition 1

*The comparison inequalities*

4.2 $$ LY ( t ) >ZH ( t ) >BS_{1} ( t ) >\max \bigl( ZS ( t ) ,Z ( t ) ,BS_{2} ( t ) \bigr) $$

*hold for* $t\in ( 0,\pi/2 ) $. *Moreover*, $ZS ( t ) $, $Z ( t ) $ *and* $BS_{2} ( t ) $ *are not comparable with each other for all* $t\in ( 0,\pi/2 ) $.

### Proof

(i) We first prove

$$D_{1} ( x ) =LY ( \sqrt{x} ) -ZH ( \sqrt{x} ) =1+\frac{1}{3p_{0}} \ln ( 1-p_{0}x ) -\frac{\pi^{2}-4x}{\pi ^{2}+ ( \pi^{2}/3-4 ) x}>0 $$

for $x\in ( 0,\pi^{2}/4 ) $. Differentiation yields

$$D_{1}^{\prime} ( x ) =-\frac{ ( 12-\pi^{2} ) ^{2}x}{27 ( 1-xp_{0} ) ( \pi^{2}+ ( \pi^{2}/3-4 ) x ) ^{2}} \biggl( x- \frac{72\pi^{2}-6\pi^{4}-9\pi^{4}p_{0}}{ ( 12-\pi^{2} ) ^{2}} \biggr) , $$

which shows that $D_{1}$ is increasing on $( 0,x_{0} ) $ and decreasing on $( x_{0},\pi^{2}/4 ) $, where

$$x_{0}=\frac{72\pi^{2}-6\pi^{4}-9\pi^{4}p_{0}}{ ( 12-\pi^{2} ) ^{2}}\approx1.75059. $$

Then we conclude that $D_{1} ( x ) >\min ( D_{1} ( 0 ) ,D_{1} ( \pi^{2}/4 ) ) =0$ with $D_{1} ( \pi^{2}/4 ) =0$ due to $p_{0}$ satisfying $\alpha_{p_{0}} ( \pi/2 ) =1$ shown in Lemma 5.

(ii) The second inequality directly follows from

$$\begin{aligned} ZH ( t ) -BS_{1} ( t ) & =\frac{\pi^{2}-4t^{2}}{\pi^{2}+ ( \pi^{2}/3-4 ) t^{2}}- \biggl( 1- \frac{4}{\pi^{2}}t^{2} \biggr) \\ & =\frac{1}{3}\frac{t^{2} ( 12-\pi^{2} ) ( \pi^{2}-4t^{2} ) }{\pi^{2} ( \pi^{2}+ ( \pi^{2}/3-4 ) t^{2} ) }>0 \end{aligned}$$

for $t\in ( 0,\pi/2 ) $.

(iii) The third one is deduced by

$$\begin{aligned}& BS_{1} ( t ) -ZS ( t ) = \biggl( 1-\frac{4}{\pi^{2}}t^{2} \biggr) -\frac{\pi^{2}-4t^{2}}{\pi^{2}+4t^{2}}=\frac{4t^{2} ( \pi^{2}-4t^{2} ) }{\pi^{2} ( \pi^{2}+4t^{2} ) }>0, \\& \frac{BS_{1} ( t ) -Z ( t ) }{\pi^{2}-4t^{2}}=\frac {1}{\pi^{2}}-\frac{ ( \pi^{2}-4t^{2} ) ^{\pi^{2}/6-1}}{ ( \sqrt{\pi^{4}+48t^{4}} ) ^{\pi^{2}/6}}>\frac{1}{\pi^{2}}- \frac { ( \pi^{2} ) ^{\pi^{2}/6-1}}{ ( \sqrt{\pi^{4}} ) ^{\pi^{2}/6} }=0, \\& BS_{1} ( t ) -BS_{2} ( t ) = \biggl( 1- \frac{4}{\pi^{2} }t^{2} \biggr) -\frac{\pi^{2}-4t^{2}-\pi^{2}t^{2}/3}{\pi ^{2}-t^{2}}=\frac {4}{\pi^{2}} \frac{t^{2} ( 12t^{2}+\pi^{4}-3\pi^{2} ) }{ ( \pi^{2}-t^{2} ) }>0, \end{aligned}$$

for $t\in ( 0,\pi/2 ) $.

(iv) Finally, we prove that $ZS ( t ) $, $Z ( t ) $ and $BS_{2} ( t ) $ are not comparable with each other for all $t\in ( 0,\pi/2 ) $. Simple computations yield

$$\begin{aligned}& \lim_{t\rightarrow0^{+}}\frac{ZS ( t ) -Z ( t ) }{t^{2}} =\lim_{t\rightarrow0^{+}}t^{-2} \biggl( \frac{\pi^{2}-4t^{2}}{\pi^{2}+4t^{2}}- \biggl( \frac{\pi^{2}-4t^{2}}{\sqrt{\pi^{4}+48t^{4}}} \biggr) ^{\pi^{2}/6} \biggr) =\frac{2}{3}\frac{\pi^{2}-12}{\pi^{2}}< 0, \\& \lim_{t\rightarrow ( \pi/2 ) ^{-}}\frac{ZS ( t ) -Z ( t ) }{\pi^{2}-4t^{2}} =\lim_{t\rightarrow ( \pi/2 ) ^{-}} \biggl( \frac{1}{\pi^{2}+4t^{2}}-\frac{ ( \pi ^{2}-4t^{2} ) ^{\pi^{2}/6-1}}{ ( \sqrt{\pi^{4}+48t^{4}} ) ^{\pi^{2}/6}} \biggr) =\frac{1}{2\pi^{2}}>0, \\& \begin{aligned} \lim_{t\rightarrow0^{+}}\frac{Z ( t ) -BS_{2} ( t ) }{t^{2}} & =\lim_{t\rightarrow0^{+}}t^{-2} \biggl( \biggl( \frac{\pi ^{2}-4t^{2}}{\sqrt{\pi^{4}+48t^{4}}} \biggr) ^{\pi^{2}/6}-\frac{\pi ^{2}-4t^{2}-\pi^{2}t^{2}/3}{\pi^{2}-t^{2}} \biggr)\\ & =-\frac{\pi ^{2}-9}{3\pi ^{2}}< 0, \end{aligned} \\& \begin{aligned} \lim_{t\rightarrow ( \pi/2 ) ^{-}} \bigl[ Z ( t ) -BS_{2} ( t ) \bigr] & = \lim_{t\rightarrow ( \pi/2 ) ^{-}} \biggl( \biggl( \frac{\pi^{2}-4t^{2}}{\sqrt{\pi^{4}+48t^{4}}} \biggr) ^{\pi^{2}/6}-\frac{\pi^{2}-4t^{2}-\pi^{2}t^{2}/3}{\pi^{2}-t^{2}} \biggr)\\ & =\frac{1}{9} \pi^{2}>0, \end{aligned} \\& \begin{aligned} ZS ( t ) -BS_{2} ( t ) & =\frac{\pi^{2}-4t^{2}}{\pi^{2}+4t^{2}}-\frac{\pi^{2}-4t^{2}-\pi^{2}t^{2}/3}{\pi^{2}-t^{2}} \\ & =\frac{1}{3}t^{2}\frac{4 ( \pi^{2}+15 ) }{ ( \pi^{2}-t^{2} ) ( \pi^{2}+4t^{2} ) } \biggl( t^{2}-\frac{\pi ^{2} ( 15-\pi^{2} ) }{4 ( 15+\pi^{2} ) } \biggr) \\ & \left\{ \textstyle\begin{array}{l@{\quad}l}< 0 & \text{if }0< t< \frac{\pi}{2}\sqrt{\frac{15-\pi^{2}}{15+\pi^{2}}},\\ >0 & \text{if }\frac{\pi}{2}\sqrt{\frac{15-\pi^{2}}{15+\pi^{2}}}< t< \frac {\pi }{2}. \end{array}\displaystyle \right . \end{aligned} \end{aligned}$$

This completes the proof. □

### Remark 2

From the above proposition we see that the sharp lower bound in (4.1) is superior to those ones given in (1.2), (1.3), (1.4), (1.5), and (1.7).

### Remark 3

Analogously, by comparing the limits at $t=0$ and $t=\pi/2$, we find the sharp upper bound in (4.1) is not comparable with those ones given in (1.2), (1.3), (1.4), (1.5), and (1.7). Here we omit all the details.

### Remark 4

We claim that the result stated in Theorem 2 is stronger than the inequality (1.8), that is, for $t\in ( 0,\pi/2 ) $, we have the inequalities

4.3 $$ 1-\frac{4}{\pi^{2}}t^{2}< 1+\frac{1}{3p_{0}}\ln \bigl( 1-p_{0}t^{2} \bigr) < \frac{t}{\tan t}< 1+ \frac{5}{2}\ln \biggl( 1-\frac{2}{15}t^{2} \biggr) < 1- \frac{t^{2}}{3} . $$

Indeed, the right hand side for this inequality in (4.3) follows from Corollary 1, while the left hand side one is the inequality connecting the first and third bounds in (4.2).

### Remark 5

Lemma 4 tells us that

4.4 $$\begin{aligned}& \frac{2}{15} < \frac{3t-3\cos t\sin t-2t\sin^{2}t}{3t^{2} ( t-\sin t\cos t ) }< \frac{4}{3\pi^{2}}\quad\text{for }t \in ( 0,\pi /2 ) , \end{aligned}$$

4.5 $$\begin{aligned}& \frac{1}{\pi^{2}} < \frac{3t-3\cos t\sin t-2t\sin^{2}t}{3t^{2} ( t-\sin t\cos t ) }< \frac{4}{3\pi^{2}}\quad\text{for }t \in ( \pi /2,\pi ) . \end{aligned}$$

Then from equation (3.7) we find that for $f^{\prime} ( t ) <0$ for $t\in ( 0,\pi ) $ when $p=1/\pi^{2}$, and so $f ( t ) < f ( 0^{+} ) =0$. This gives the following inequality:

$$\frac{t\cos t}{\sin t}-1-\frac{\pi^{2}}{3}\ln \biggl( 1-\frac{t^{2}}{\pi ^{2}} \biggr) < 0 $$

for all $t\in ( 0,\pi ) $, which can be stated as the following proposition.

### Proposition 2

*For all* $t\in ( 0,\pi ) $, *we have*

$$\frac{t}{\tan t}< 1+\frac{\pi^{2}}{3}\ln \biggl( 1-\frac{t^{2}}{\pi^{2}} \biggr) . $$

### Remark 6

The inequality

$$\frac{\sin t}{t}< \frac{2+\cos t}{3},\quad t\in \biggl( 0,\frac{\pi }{2} \biggr), $$

is true due to Cusa and Huygens’ paper (see, *e.g.* [19]), which is now known as *Cusa’s inequality* (see *e.g.* [8, 20–23]). Some refinements and generalizations of *Cusa’s inequality can be found in* [8, 21, 22, 24–29]. Now by letting $t=x/2$ and simplifying, inequalities (4.4) and (4.5) can be written as

$$\begin{aligned}& \frac{x ( x-\sin x ) }{30} < \frac{2+\cos x}{3}-\frac{\sin x}{x}< \frac{x ( x-\sin x ) }{3\pi^{2}}\quad\text{for }x\in ( 0,\pi ) , \\& \frac{x ( x-\sin x ) }{4\pi^{2}} < \frac{2+\cos x}{3}-\frac {\sin x}{x}< \frac{x ( x-\sin x ) }{3\pi^{2}}\quad\text{for }x\in ( \pi,2\pi ) , \end{aligned}$$

which give stronger versions of Cusa’s inequality.

### Proposition 3

*We have*

4.6 $$\begin{aligned}& \frac{2+\cos x}{3}-\frac{x ( x-\sin x ) }{3\pi^{2}} < \frac {\sin x}{x}< \frac{2+\cos x}{3}-\frac{x ( x-\sin x ) }{30}\quad\textit {for }x\in ( 0,\pi ) , \end{aligned}$$

4.7 $$\begin{aligned}& \frac{2+\cos x}{3}-\frac{x ( x-\sin x ) }{3\pi^{2}} < \frac {\sin x}{x}< \frac{2+\cos x}{3}-\frac{x ( x-\sin x ) }{4\pi^{2}}\quad \textit{for }x\in ( \pi,2\pi ) . \end{aligned}$$

*Moreover*, *the two double inequalities are sharp*.

### Remark 7

In [12], Corollary 12, Yang *et al.* proved that, for $t\in ( 0,\pi/2 ) $,

$$\sqrt{\exp \biggl( \frac{t}{\tan t}-1 \biggr) }< \frac{\sin t}{t}< \exp \biggl( \frac{t}{\tan ( t/2 ) }-2 \biggr) . $$

Then by inequalities (3.10) for $p=4/ ( 3\pi^{2} ) $ and $q=2/15$, we obtain

$$\biggl( 1-\frac{4t^{2}}{3\pi^{2}} \biggr) ^{\pi^{2}/8}< \sqrt{\exp \biggl( \frac{t}{\tan t}-1 \biggr) }< \frac{\sin t}{t}< \exp^{2} \biggl( \frac{t/2}{\tan ( t/2 ) }-1 \biggr) < \biggl( 1-\frac{t^{2}}{30} \biggr) ^{5} $$

for $t\in ( 0,\pi/2 ) $. Further, the right hand side inequalities can be improved as follows.

### Proposition 4

*The inequalities*

4.8 $$ \begin{aligned}[b]\rho_{r} \bigl( 1-rt^{2} \bigr) ^{1/ ( 6r ) }&< \lambda_{s}\exp \biggl( \frac{st}{\tan ( st ) }-1 \biggr) \\ &< \frac{\sin t}{t}< \exp \biggl( \frac{st}{\tan ( st ) }-1 \biggr) < \bigl( 1-rt^{2} \bigr) ^{1/ ( 6r ) }\end{aligned} $$

*hold for* $t\in ( 0,\pi/2 ) $ *with the best constants* $s=1/\sqrt {2}$, $r=1/15$ *and*

$$\begin{aligned}& \lambda_{s} =\frac{2}{\pi\exp ( \sqrt{2}\pi\cot ( \sqrt{2}\pi/4 ) -1 ) }\approx0.99801, \\& \rho_{r} =\frac{2}{\pi ( 1-\pi^{2}/60 ) ^{5/2}}\approx 0.99771. \end{aligned}$$

### Proof

Let

$$h ( t ) =\frac{st\cos ( st ) }{\sin ( st ) }-1-\ln\frac{\sin t}{t}, $$

where $s=1/\sqrt{2}$. Differentiation yields

$$h^{\prime} ( t ) =\frac{1}{t}-\frac{\cos t}{\sin t}+s \frac{\cos st}{\sin st}-s^{2}\frac{t}{\sin^{2}st}. $$

Expanding in power series leads to

$$h^{\prime} ( t ) =\sum_{n=1}^{\infty} \bigl( 1-2ns^{2n} \bigr) \frac{2^{2n}}{ ( 2n ) !}|B_{2n}|t^{2n-1}= \sum_{n=1}^{\infty } \bigl( 2^{n}-2n \bigr) \frac{2^{n}}{ ( 2n-1 ) !}|B_{2n}|t^{2n-1}>0. $$

This indicates that $h ( \pi/2 ) >h ( t ) >h ( 0^{+} ) =0$ for $t\in ( 0,\pi/2 ) $, which proves the second and third inequalities of (4.8). Considering the limit

$$\lim_{t\rightarrow0}\frac{h ( t ) }{t^{2}}=\lim_{t\rightarrow 0} \frac{\frac{st\cos ( st ) }{\sin ( st ) }-1-\ln \frac{\sin t}{t}}{t^{2}}=\frac{1}{3} \biggl( \frac{1}{2}-s^{2} \biggr) , $$

it is seen that $s=1/\sqrt{2}$ and $\lambda_{s}$ are the best possible constants.

The first and fourth ones are derived from the decreasing property of $f ( st ) \equiv f ( u ) $ for $u\in ( 0,s\pi/2 ) \subset ( 0,\pi/2 ) $ proved in Theorem 1 for $p=r/s^{2}=2/15$, and then $r=1/15$ and $\rho_{r}$ are also the best. This completes the proof. □

## Applications

In this section, we give some precise estimations for the Sine integral and Catalan constant. The Sine integral is defined by

$$\operatorname*{Si} ( x ) = \int_{0}^{x}\frac{\sin t}{t}\,dt. $$

There are many interesting results concerning the Sine integral; see [26, 30–33] and the references therein. Now we shall give more accurate estimations.

### Proposition 5

*For* $x\in(0,\pi]$, *we have*

5.1 $$ \frac{2x+\sin x}{3}-\frac{x^{3}+3x\cos x-3\sin x}{9\pi ^{2}}< \operatorname{Si} ( x ) < \frac{2x+\sin x}{3}-\frac{x^{3}+3x\cos x-3\sin x}{90}. $$

*In particular*, *we have*

$$\begin{aligned}& 0.75897 \approx\frac{95\pi}{576}+\frac{4\pi^{2}-\pi+4}{24\pi^{2}}\sqrt{2}< \operatorname{Si} \biggl( \frac{\pi}{4} \biggr) < \frac{\pi ( 960-\pi^{2} ) }{5760}+ \frac{44-\pi}{240}\sqrt{2}\approx0.75898, \\& 1.3707 \approx\frac{23\pi^{3}+24\pi^{2}+24}{72\pi^{2}}< \operatorname{Si} \biggl( \frac{\pi}{2} \biggr) < \frac{264+240\pi-\pi^{3}}{720}\approx 1.3708, \\& 1.8514 \approx\frac{5\pi^{2}+3}{9\pi}< \operatorname{Si} ( \pi ) < \frac{\pi ( 63-\pi^{2} ) }{90} \approx1. 8546. \end{aligned}$$

### Proof

Indeed, integrating both sides over $[ 0,x ] $ for double inequality (4.6) easily yields (5.1). Direct computations give the approximation values of $\operatorname{Si} ( x ) $ for $x=\pi/4, \pi/2, \pi$. □

Note that

$$\int_{0}^{\pi/2}\ln ( \sin x )\, dx=-\frac{\pi}{2} \ln2\approx -1.08879 $$

and

5.2 $$ \int_{0}^{x}\frac{t}{\tan t}\,dt=x\ln ( \sin x ) - \int_{0}^{x}\ln ( \sin t )\, dt. $$

We are now in the position to evaluate the integral $\int_{0}^{x}\ln ( \sin t )\, dt$ for $x\in ( 0,\pi/2 ) $.

### Proposition 6

*Let* $x\in ( 0,\pi/2 ) $. *Then*, *for* $p\in ( 0,2/15 ) $, *we have*

5.3 $$ L_{p} ( x ) < \int_{0}^{x}\ln ( \sin t )\, dt< U_{p} ( x ) , $$

*where*

$$\begin{aligned}& L_{p} ( x ) =x\ln ( \sin x ) -\frac{3p-2}{3p}x- \frac{1}{3p}x\ln \bigl( 1-px^{2} \bigr) -\frac{1}{3p^{3/2}}\ln \frac {\sqrt{p}x+1}{1-\sqrt{p}x}, \\& U_{p} ( x ) =x\ln ( \sin x ) -\frac{x^{2}}{\tan x}+ \frac{2}{3p}x-\frac{1}{3p^{3/2}}\ln\frac{\sqrt{p}x+1}{1-\sqrt{p}x}. \end{aligned}$$

*The double inequality* (5.3) *is reversed for* $p\in [ 4/ ( 3\pi^{2} ) ,4/\pi^{2} ] $. *In particular*, *we have*

5.4 $$ x\ln ( \sin x ) -x+\frac{1}{9}x^{3}< \int_{0}^{x}\ln ( \sin t ) \,dt< x\ln ( \sin x ) - \frac{x^{2}}{\tan x}-\frac{2}{9}x^{3}. $$

### Proof

By the proof of Theorem 1 we see that the function

$$t\rightarrow\ln Y ( t ) -\ln B_{p} ( t ) =\frac {t}{\tan t}-1- \frac{1}{3p}\ln \bigl( 1-pt^{2} \bigr) $$

is strictly decreasing on $( 0,\pi/2 ) $ if $p\leq2/15$ and increasing on $( 0,\pi/2 ) $ if $4/ ( 3\pi^{2} ) \leq p\leq4/\pi^{2}$. Then, for $t\in(0,x]\subset ( 0,\pi/2 ) $, we have, for $p\leq2/15$,

$$\frac{x}{\tan x}-1-\frac{1}{3p}\ln \bigl( 1-px^{2} \bigr) \leq\frac {t}{\tan t}-1-\frac{1}{3p}\ln \bigl( 1-pt^{2} \bigr) < 0, $$

that is,

$$\frac{x}{\tan x}-\frac{1}{3p}\ln \bigl( 1-px^{2} \bigr) + \frac{1}{3p}\ln \bigl( 1-pt^{2} \bigr) \leq \frac{t}{\tan t}< 1+\frac{1}{3p}\ln \bigl( 1-pt^{2} \bigr) , $$

which is the reverse for $4/ ( 3\pi^{2} ) \leq p\leq4/\pi^{2}$.

Integrating both sides over $[ 0,x ] $ gives, for $p\in ( 0,2/15 ) $,

$$\begin{aligned} & \frac{x^{2}}{\tan x}-\frac{x}{3p}\ln \bigl( 1-px^{2} \bigr) + \frac{1}{3p} \biggl( x\ln \bigl( 1-px^{2} \bigr) + \frac{1}{\sqrt{p}}\ln\frac{\sqrt {p}x+1}{1-\sqrt{p}x}-2x \biggr) \\ &\quad \leq \int_{0}^{x}\frac{t}{\tan t}\,dt< x+ \frac{1}{3p} \biggl( x\ln \bigl( 1-px^{2} \bigr) + \frac{1}{\sqrt{p}}\ln\frac{\sqrt{p}x+1}{1-\sqrt{p}x}-2x \biggr) . \end{aligned}$$

Combining with equation (5.2) gives the double inequality (5.3) for $p\in(0,2/15]$.

Moreover, it is clear that the double inequality (5.3) is reversed if $4/ ( 3\pi^{2} ) \leq p\leq4/\pi^{2}$.

Letting $p\rightarrow0^{+}$ in (5.3) yields (5.4), which completes the proof. □

### Remark 8

Taking $p=4/ ( 3\pi^{2} ) $ and $x=\pi/2, \pi/4$ in the double inequality (5.3) and computing give

$$\begin{aligned}& L_{4/ ( 3\pi^{2} ) } \biggl( \frac{\pi}{2} \biggr) =-\pi \frac{\pi^{2}\ln2-\pi^{2}\ln3+\sqrt{3}\pi^{2}\ln ( 2+\sqrt{3} ) -2\pi^{2}+4}{8} \approx-1.08854, \\& U_{4/ ( 3\pi^{2} ) } \biggl( \frac{\pi}{2} \biggr) =-\pi ^{3} \frac{\sqrt{3}\ln ( \sqrt{3}+2 ) -2}{8}\approx-1.08924, \\& \begin{aligned} &L_{4/ ( 3\pi^{2} ) } \biggl( \frac{\pi}{4} \biggr)\\ &\quad =-\pi \frac{2\sqrt{3}\pi^{2}\ln ( 13+4\sqrt{3} ) -\pi^{2}\ln12-\pi ^{2} ( 2\sqrt{3}-1 ) \ln11-2\pi^{2}+2\ln2+4}{16}\\ &\quad \approx -1.00236, \end{aligned}\\& \begin{aligned}U_{4/ ( 3\pi^{2} ) } \biggl( \frac{\pi}{4} \biggr) &=-\pi \frac{2\sqrt{3}\pi^{2}\ln ( 4\sqrt{3}+13 ) -2\sqrt{3}\pi^{2}\ln11-2\pi^{2}+\pi+2\ln2}{16}\\ &\approx-1.00243. \end{aligned} \end{aligned}$$

Then we obtain

5.5 $$\begin{aligned}& -1.08924 \approx U_{4/ ( 3\pi^{2} ) } \biggl( \frac{\pi}{2} \biggr) < \int_{0}^{\pi/2}\ln ( \sin t ) \,dt< L_{4/ ( 3\pi^{2} ) } \biggl( \frac{\pi}{2} \biggr) \approx -1.08854, \end{aligned}$$

5.6 $$\begin{aligned}& -1.00243 \approx U_{4/ ( 3\pi^{2} ) } \biggl( \frac{\pi}{4} \biggr) < \int_{0}^{\pi/4}\ln ( \sin t )\, dt< L_{4/ ( 3\pi^{2} ) } \biggl( \frac{\pi}{4} \biggr) \approx-1.00236. \end{aligned}$$

Clearly, the absolute errors of the two approximations are less than 0.0007 and 0.00007.

It is well known that the Catalan constant appearing in [34–36]

$$G=\sum_{n=0}^{\infty}\frac{ ( -1 ) ^{n}}{ ( 2n+1 ) ^{2}}=0.9159655941772190\ldots $$

is a famous mysterious constant appearing in many places in mathematics and physics. Its integral representations [37] include the following:

$$\begin{aligned} G & = \int_{0}^{1}\frac{\arctan x}{x}\,dx= \frac{1}{2} \int_{0}^{\pi/2}\frac {x}{\sin x}\,dx \\ & =-2 \int_{0}^{\pi/4}\ln ( 2\sin x )\, dx= \frac{\pi^{2}}{16}-\frac{\pi}{4}\ln2+ \int_{0}^{\pi/4}\frac{x^{2}}{\sin^{2}x}\,dx. \end{aligned}$$

Now, by using the third integral representation for *G* and (5.6), we easily obtain a very accurate approximation for *G*, the absolute error of which is less than 0.00015.

### Proposition 7

*We have*

$$0.91594\approx-\frac{\pi}{2}\ln2-2L_{4/ ( 3\pi^{2} ) } \biggl( \frac{\pi}{4} \biggr) < G< -\frac{\pi}{2}\ln2-2U_{4/ ( 3\pi^{2} ) } \biggl( \frac{\pi}{4} \biggr) \approx0.91608. $$

### Remark 9

Clearly, the above estimate for *G* is superior to Yang’s presentation in [26], Proposition 4, [33], Remark 4.2.

## Conclusions

Rather than using classical approaches, we in this paper presented the new upper and lower bounds of $\frac{\tan t}{t}$ on the interval $(0,\pi/2 )$ by way of the monotonicity criterion for the quotient of power series. Our conclusions have not only refined the Redheffer and Becker-Stark type inequalities concerning the tangent function, but they also showed some more precise estimations to the Sine integral and the Catalan constant. More precisely, our conclusion is that the sharp lower bound of $\frac{\tan t}{t}$ is superior to all given results as showed by Proposition 1 in Section 4, although its sharp upper bound is not comparable with those given ones. In addition, we also derived a stronger version of Cusa’s inequality, and a very accurate approximation of the Catalan constant with the absolute error being less than 0.00015.
